# Biodegradable and Sustainable Synthetic Antibodies—A Perspective

**DOI:** 10.3390/pharmaceutics15051440

**Published:** 2023-05-09

**Authors:** Xiaohan Ma, Jonathan C. Knowles, Alessandro Poma

**Affiliations:** 1 Division of Biomaterials and Tissue Engineering, UCL Eastman Dental Institute, Royal Free Hospital, UCL Medical School, Rowland Hill Street, London NW3 2PF, UK; 2 Department of Nanobiomedical Science and BK21 PLUS NBM Global Research Center for Regenerative Medicine, Dankook University, Cheonan 31116, Republic of Korea; 3 UCL Eastman-Korea Dental Medicine Innovation Centre, Dankook University, Cheonan 31116, Republic of Korea

**Keywords:** molecular imprinting, molecularly imprinted polymers, nanoparticles, synthetic antibodies, biodegradable polymers, sustainable imprinting, sustainability

## Abstract

Molecular imprinting technology has been around for almost a century, and we have witnessed dramatic advancements in the overall design and production of molecularly imprinted polymers (MIPs), particularly in terms of possible formats of the final products when it comes to truly resembling antibody substitutes, i.e., MIP nanoparticles (MIP NPs). Nonetheless, the overall technology appears to struggle to keep up with the current global sustainability efforts, as recently elucidated in the latest comprehensive reviews, which introduced the “GREENIFICATION” concept. In this review, we will try to elucidate if these advancements in MIP nanotechnology have indeed resulted in a sustainability amelioration. We will do so by discussing the general production and purification strategies for MIP NPs, specifically from a sustainability and biodegradation perspective, also considering the final intended application and ultimate waste management.

## 1. Introduction

The expression “molecular recognition” has seen increased use since the early 1980s, specifically indicating a group of phenomena driven by *ad hoc* physicochemical interactions (mainly noncovalent) [1]. These phenomena are critical for certain biological processes (e.g., ligand-receptor interactions), and a significant proportion of current research in chemistry, materials as well as pharmaceutical areas is indeed driven by the possibility of designing and tailoring specific molecular interactions suitable to be successfully employed in the desired applications [1,2,3,4,5].

**Figure 1:**
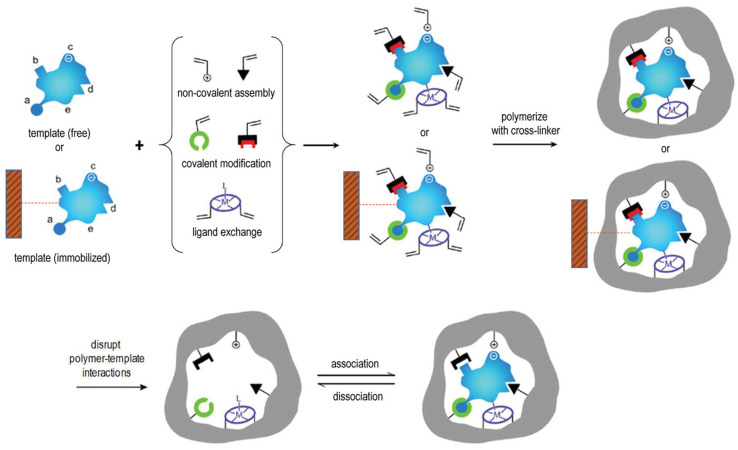
Scheme of the molecular imprinting process: the establishment of interactions between the template (free in solution or immobilized on a suitable solid support) and polymerizable groups interacting either covalently (a,b), non-covalently (c,d), or *via* co-ordination with a metal center (e) with suitable functional groups or structural elements of the template. Subsequent polymerization in presence of a cross-linker develops a porous insoluble matrix containing the binding sites for the template. At this point, either the template is removed (if free), or alternatively, the polymer is separated from the immobilized template in suitable washing/elution conditions. In all cases, the target analyte can selectively rebind to the polymer into the sites formed by the template or “imprints”. Reproduced with permission from Patel *et al*. [10].

Amongst these applications, there has been an entire research avenue being developed since the 1930s to implement dedicated molecular recognition properties into materials, more specifically in polymers [6]. This process is known as *molecular imprinting*, and the related polymeric products are called *molecularly imprinted polymers* (or MIPs). MIPs are generated around a target species (or *template*) by exploiting mainly (but not exclusively) non-covalent interactions between the said template (which could be a small molecule, a peptide, a protein or even a whole cell) and the building blocks of the polymeric material. MIPs are normally generated using a free-radical polymerization or a polycondensation process. At the end of the reaction, the template is then washed away from the final polymeric product, which can be then processed further depending on the intended application (e.g., separation, sensing, etc.) [7,8,9] (Figure 1).

**Figure 2:**
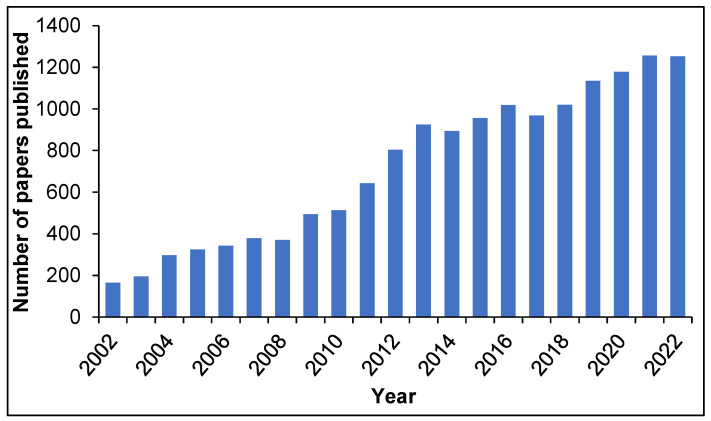
Number of published papers on molecular imprinting in the years 2002–2022. Source: Scopus.

MIPs nowadays are indeed an established technology, as demonstrated by the exponentially increasing number of patents filed and papers published yearly on the topic (Figure 2).

**Figure 3:**
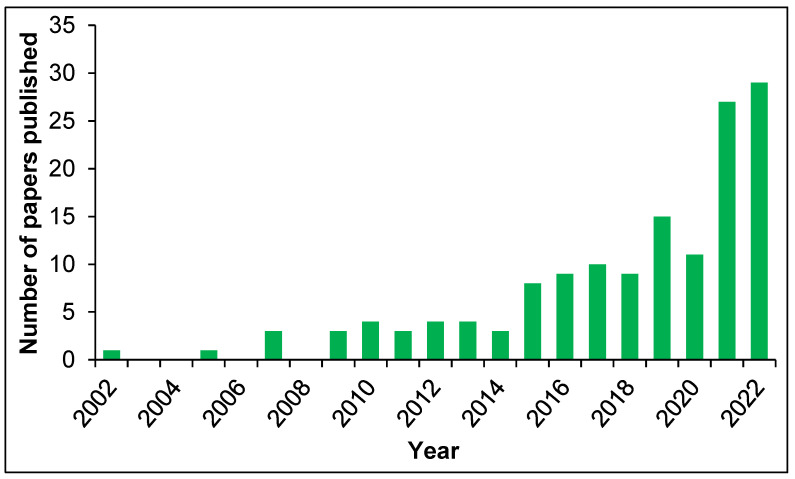
Number of published papers on molecular imprinting and sustainability/biodegradability in the years 2002–2022. Source: Scopus (Keywords searched: “molecular imprinting” AND “sustainable” OR “biodegradable”).

When it comes to sustainability, it is a common opinion that the technology, however, is really not up to date [11,12]. Indeed, in comparison to the general number of MIPs papers published throughout the years, the same search corrected by adding terms related to the sustainability/biodegradability topic does not provide corresponding numbers (Figure 3).

**Figure 4:**
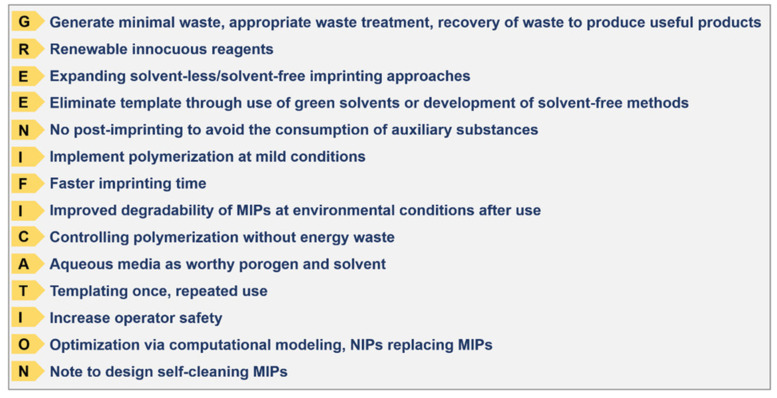
The fourteen principles of green molecular imprinting expressed as the mnemonic device “GREENIFICATION.” Reproduced with permission from Arabi *et al*. [11].

First of all, as previously mentioned, the vast majority of molecularly imprinted polymers are made of acrylic or methacrylic monomers and cross-linkers, which eventually results in a material that is inherently not biodegradable. Moreover, many of the synthetic processes heavily rely on the use of vast volumes of organic solvents, not only for the synthesis but also for the purification and template extraction steps. Furthermore, in classical molecular imprinting, a new template is consumed with every new synthetic process, which represents a significant burden not only in terms of a lack of recycling but also from a financial perspective (since normally the template represents the most expensive component of the whole production process). Last but not least, monomer and solvent waste also represent an important sustainability aspect which needs addressing, because especially in certain types of synthesis strategies (e.g., high-concentration controlled polymerization) many of the monomers do not actually end up in the final material, but are discarded as waste. This is a far from comprehensive summary of the aspects that currently render MIP technology poorly sustainable. For a more comprehensive discussion on this, we would like to refer the readers to the “GREENIFICATION” work on MIP technology devised by Arabi *et al*. [11,12] (Figure 4).

Nevertheless, over the past 20 years, the overall trend of the production of MIPs has shifted towards the generation of a more “biomimetic” format, i.e., nanoparticles (MIP NPs), which better resemble the natural antibody counterparts and can be truly defined as *synthetic antibodies* [8,13,14]. From a “green” point of view, though, it is not clear yet if this paradigm shift has indeed resulted in a sustainability amelioration. In this review, we will try to elucidate this particular issue by discussing the general production and purification strategies for MIP NPs, specifically from a sustainability perspective, and also consider the ultimate waste management. We will not go into detail about the applications of MIP NPs, and for this, we refer the reader to recent and more comprehensive reviews [8,10,15,16,17,18,19]. Our final aim is to understand whether the current research avenues are truly leading towards the generation of biodegradable and sustainable synthetic antibodies.

## 2. MIP NPs Production

### 2.1. Polymerization Chemistry

**Figure 5:**
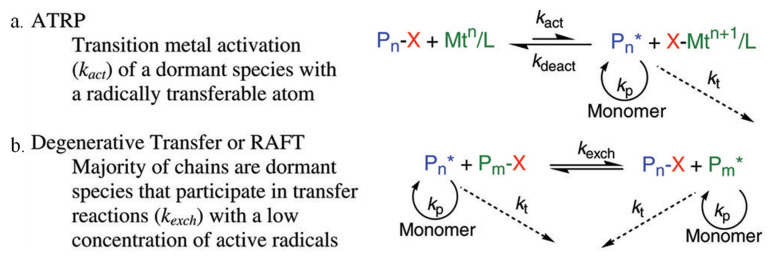
ATRP (**a**) and RAFT (**b**) controlled radical polymerization methods. Adapted with permission from Matyjaszewski and Spanswick [24].

Before delving into the specific methods of MIP NPs manufacturing, it is important to distinguish the different chemical processes at the base of these methods. Independently from the type of manufacturing technology, the majority of MIP NPs are produced *via* either thermally, photo- or redox-initiated free radical polymerization. The popularity of this chemistry relies mainly on its flexibility and compatibility with a high number of functional monomers and reaction conditions, coupled with an extreme simplicity of execution [20,21]. Nonetheless, a number of groups have successfully employed more controlled radical polymerization methods, such as atom transfer radical polymerization (ATRP) or reversible addition-fragmentation chain transfer (RAFT) polymerization [22,23]. The main advantage of these techniques is the control they offer on the final polymeric product (Figure 5), although this is more relevant in the context of producing more complex polymer architectures.

From a sustainability perspective, in general, radical polymerization processes heavily rely on the use of organic solvents, and therefore the generation of solvent and chemical waste (especially in the case of large-scale synthesis) is significant. Moreover, thermal and photo initiation are heavily energy-consuming, and risks to the health and safety of the operators and users might arise from the generation of hazardous chemical gases and/or exposure to UV or toxic chemicals [11,12]. Furthermore, thinking ahead about the biodegradability component, independently from the amount of control achieved on the polymerization process, these methods end up producing a carbon-carbon backbone, which is inherently non-biodegradable (*vide infra*).

Another type of polymerization process used to prepare imprinted NPs is polycondensation, specifically in the case of silica- or titania-based MIP NPs [25]. These materials are more environmentally friendly than polymethacrylates and polyacrylates, for several reasons. First, the polymerization reaction can take place in “green” solvents, such as water or ethanol, without the need for extensive heating or other forms of energy control, whilst the final properties of the imprinted material can be finely tuned by adjusting the drying conditions [26,27]. In addition, imprinted silica and titania are low-cost materials and exhibit low reactivity and swelling (even in harsh conditions), and can withstand aging [26,28]. Nonetheless, these materials and their preparation are burdened by some disadvantages. First of all, some metal alkoxide molecular precursors and monomers (e.g., tetraethyl orthosilicate and (3-aminopropyl)triethoxysilane, which are widely used in silica MIPs) are highly toxic. Secondly, although high-surface silicon NPs are considered biodegradable, the large-scale disposal of these materials requires extreme heat [29,30]. 

**Figure 6:**
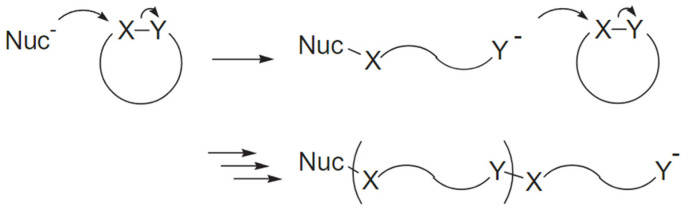
Generic ring−opening polymerizations (ROP) scheme using a nucleophilic initiator (Nuc^−^). The polarized functional group in cyclic monomers is represented by X−Y. The ring−opening reaction of the monomer is triggered by a nucleophilic attack of the initiator to the atom X, with the release of Y^−^, which in turn will continue to attack the atom X in another monomer. Adapted with permission from Endo [40].

The holy grail of molecular imprinting polymerization technology from a sustainability point of view would be to exploit the synthesis of materials which are wholly biodegradable, possibly derived from sources “generally recognized as safe” (GRAS) [31,32]. These materials, for example, could be fully bio-based polymers (e.g., polydopamine, chitosan, gelatin) [33,34,35,36] or even polyesters or polyaminoacids that are usually prepared *via* ring-opening polymerization (ROP) (e.g., of lactide, caprolactone, glycolide, etc.) [21,37,38,39] (Figure 6).

Although this strategy results in extremely biodegradable and environmental-friendly polymers, unfortunately it is not very compatible with molecular imprinting due to the susceptibility of the polymerization process to protic groups, which instead would be necessary to guarantee a suitable imprinting effect. Although some strategies have been developed that highlight the possibility of “tolerance” to protic groups [41,42,43], these are quite limited and currently no reports of pure ROP have been highlighted for molecular imprinting.

### 2.2. Manufacturing Methods

#### 2.2.1. Bulk Polymerization

MIPs are classically produced *via* polymerization in bulk, where a high concentration of monomers in a suitable solvent is polymerized extensively until a monolith is obtained [23,44,45,46,47,48,49]. Historically, these monoliths were then processed by grinding and fractionated *via* sieving to collect a regularly-sized powder which could then be exploited for further applications. This method is indeed still used, especially when quick and easy tests might be required (e.g., to assess whether a polymerization mixture would be suitable for a certain target) [46,49]. Nonetheless, it is extremely poorly compatible with the generation of MIP NPs. It would entail the exploitation of a full “top-down” strategy with the isolation of a nanomaterial from a “macro” monolithic starting point. To the best of our knowledge, such a method has only been used once to generate MIP NPs with limited success and yield and extensive processing and purification were required [50]. From a sustainability point of view, whilst classical bulk polymerization minimizes the waste in relation to the mass of the obtained product, this is not the case when it is used to produce MIP NPs since the majority of the “bulk” powder is discarded and only the colloidal fraction is collected. Furthermore, this is normally achieved via ultrafiltration, e.g., via disposable centrifugal devices, which would then add to the overall environmental impact of the whole method, rendering it poorly sustainable for the production of MIP NPs [50].

#### 2.2.2. Precipitation Polymerization

**Figure 7:**
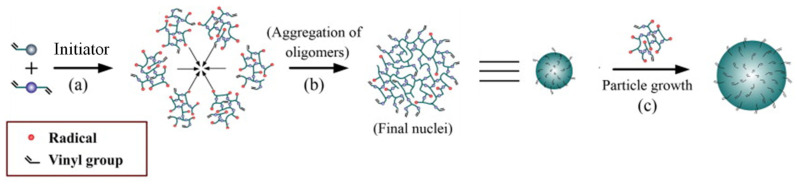
Schematic of the formation of nanoparticles during precipitation polymerization: (**a**,**b**) particle nucleation, (**c**) particle growth. Adapted with permission from Zhang *et al*. [51].

Precipitation polymerization is amongst the most used methods to prepare MIP NPs in all of its variations. It is efficient, simple and relatively rapid in comparison to other more complex methods (such as emulsion approaches). The key to a successful precipitation polymerization process is to perform the process in adequately calculated diluted conditions (~2% *v*/*v* monomer concentration) where the monomers are soluble but the polymer colloidal nanomaterials are not, therefore resulting in a coacervation process where MIP NPs are produced and can be isolated [7,51] (Figure 7).

The challenge intrinsic to this method in its more conventional form is mainly related to the high amount of solvents required for production and/or purification, as well as template consumption [7,13]. Furthermore, it is paramount to control the physico-chemical parameters of the reaction in terms of temperature and stirring, therefore, resulting in a significant energy consumption level. Last but not least, the classical form of the method is quite time-consuming [7,13].

To offset some of these aspects, Ye and his group in 2009 pioneered what is called distillation precipitation polymerization, where the polymerization process and MIP NPs formation are carried out in reflux conditions whilst removing the solvent. This results in a gradual increase in the concentration of the reagents, which ultimately accelerates the process, thus allowing to obtain MIP NPs in as little as 30 min [52,53]. Therefore, although the energy consumption is high to maintain the distillation process, this is only for a short amount of time. Furthermore, waste production is significantly reduced since the majority of the initial solvent can be recovered from the collection vessel and possibly recycled. Even though it appears an advantageous process from a sustainability perspective, the high temperature makes it incompatible with thermolabile templates or even monomers. Furthermore, the use of such high temperatures during the imprinting process can compromise the affinity and selectivity of the final products [47,52,54].

For compatible templates and monomers, some of the disadvantages listed above can be addressed by shifting the whole polymerization process in water, using redox-, photo- or enzyme-initiation [55,56,57]. Although a surfactant was used to stabilize the growing NPs in the early stages of the optimization for this method, it was observed that this was not essential (provided that a certain increase in polydispersity can be deemed acceptable in the final product). Furthermore, avoiding the use of a surfactant extremely facilitates the purification process. Thanks to its simplicity and mild conditions, there have been numerous examples reported in the literature of MIP NPs produced for small molecules as well as biomacromolecules [58,59,60]. From a sustainability perspective, though, it has to be highlighted that template consumption can be significant as well as waste production for the purification process (which is normally *via* extensive and lengthy dialysis).

#### 2.2.3. Early-Termination Polymerization

The so-called early-termination polymerization is similar but at the same time opposite to precipitation polymerization. The principle is shared between bulk polymerization and precipitation polymerization. In a sense, the polymerization process takes place at a high concentration of monomers and templates, but it is stopped in the very early stages so to produce nanogels instead of bulk polymers.

This has been achieved by Wulff *et al*. using a “post-dilution” approach, where the polymerization was terminated early by massively diluting the solution [61,62]. From a sustainability perspective, this approach seems quite compatible with large-scale purposes, thanks to the high yields obtained of relatively monodisperse MIP NPs. Unfortunately, there is a significant amount of solvent and monomer/template waste to deal with at the end of the process. Furthermore, the biggest hurdle is related to the fact that, although the initial polymerization at high concentration is only carried out for 2 h, the authors performed a further step-wise thermal polymerization process in diluted solution for almost 2 weeks, with temperatures ranging from 60 to 80 °C. This renders the process amongst the least sustainable and green for producing MIP NPs.

**Figure 8:**
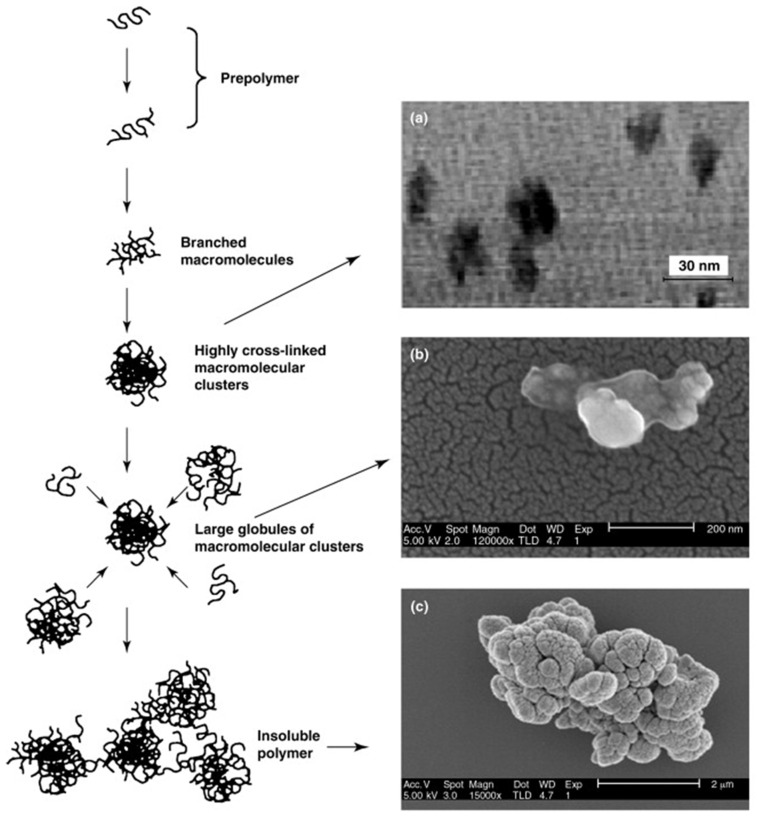
Schematic of the early-termination of UV-initiated iniferter polymerization. Monomers begin to combine from solution to form small polymer chains, which progressively increase their branching degree. Highly cross-linked macromolecular clusters are then formed, and on further reactions, these clusters bind to each other, giving rise to globules and eventually to the insoluble polymer. (**a**) TEM image of nanoparticles formed by 170 s of UV irradiation (magnification 340,000×). (**b**,**c**) SEM images of polymers formed by aggregation of molecular clusters achieved during 180 and 250 s of irradiation, respectively. Adapted with permission from Piletsky *et al*. [63].

Another option has been pioneered by the group of Piletsky, who reported an early-termination of a UV-initiated iniferter polymerization [63] (Figure 8).

The particular “start-stop” properties of the iniferter when exposed to UV radiation allowed them to perform an imprinting polymerization process at a high concentration for a few minutes without resulting in the production of bulk monoliths [64,65,66]. The overall energy consumption is minimal, but probably the major drawback in terms of sustainability is related to the unused monomer waste. Indeed, the process has an extremely low yield since the majority of the monomers and templates are discarded as waste during the purification.

#### 2.2.4. Mini-Emulsion Polymerization

An effective and popular method to produce MIP NPs in high yield is mini-emulsion polymerization. As for any emulsification process, a water and oil phase require mixing in the presence of one or more surfactants to stabilize the droplets [67]. To obtain MIP NPs, a high-shear homogenization step is required, thus resulting in droplets and subsequent MIP NPs of diameters ranging from 30 to 500 nm [48,68,69]. There are many advantages to the method, including the ease of handling, mild polymerization conditions, a high potential for mass production and small and uniform particle size.

Different types of surfactants can be used for the preparation, and they can also be exploited to confine imprinting to the surface of synthesized MIP NPs [70,71]. Nonetheless, even if mini-emulsion polymerization can produce very small spherical nanoparticles, the presence of surfactants can have a significant effect not only on the product (in terms of affinity and selectivity) but also on the overall sustainability and green properties of the process, ultimately increasing the negative impact on the environment. Even after multiple rinsing steps, residual surfactant molecules remain on the MIP NPs and can interfere with the recognition process. More importantly, the presence of detergents renders this production method virtually unsuitable to imprint proteins, which would be denatured by the surfactant [72].

The environmental risk of surfactants can be mitigated by the use of biosurfactants, such as rhamnolipids, as a green and renewable alternative [11,44]. Alternatively, a number of examples have been reported in the literature of “Pickering” emulsions, where the stabilizer is a solid particle (e.g., silica [73,74] or chitosan NPs [75]), although mainly to produce MIP microparticles. The energy consumption for the high-shear homogenization, as well as the overall waste produced during the purification steps, need to be taken into consideration in addition to the surfactant when evaluating the overall green properties of the process.

#### 2.2.5. Solid-Phase Synthesis

A recent strategy developed to synthesize MIP NPs is solid-phase synthesis. It can be considered a flexible synthetic strategy, potentially adaptable to most of the processes discussed thus far (although it is normally favored for precipitation polymerization and early termination polymerization). It was pioneered by the group of Piletsky in 2013 and it involves the immobilization of the template on a suitable solid phase (e.g., glass microspheres or magnetic nanoparticles) which can then double as an affinity separation matrix to actually select the “very best” MIP NPs with high affinity and specificity for the target, removing the low-affinity fraction [13,76]. Despite being an advantageous process, also thanks to its possibility of full automation, depending on the strategy used (e.g., aqueous polymerization or early termination of iniferter-initiated polymerization), there is a significant amount of waste produced and yields do not normally exceed 30 to 40% in weight depending on the polymerization process [13,77]. 

Furthermore, it adds a new type of solid waste to the equation. Nonetheless, many examples have been published, demonstrating that this solid phase bearing the template can actually be recycled multiple times without apparent loss of performance and, depending on the chemistry, it could potentially be possible to actually regenerate the binding surface to exchange the immobilized template for another one [14]. Nonetheless, more systematic studies are required to actually explore the boundaries of the technique from a sustainability perspective.

## 3. Biocompatibility and Biodegradability

When considering potential exposure risks as well as healthcare applications, and also taking into account the environmental risks of the synthesized products, we strongly believe that it should be paramount nowadays to consider the biocompatibility and biodegradability characteristics of MIP NPs. As initially discussed, the majority of imprinted polymers and, therefore, also MIP NPs, are actually built on a carbon-carbon backbone which is inherently not prone to degradation. Taking into consideration the importance of the build-up and accumulation of non-biodegradable plastic in the environment, to the point that microplastics have been found in a number of water bodies as well as glaciers and water organisms [78,79], it would be foolish to consider a large-scale introduction of MIP nanotechnology into the world without considering the potential environmental implications. Fortunately, a number of studies have currently reported the effects of MIP NPs *in vivo* [80,81,82,83,84], in certain cases assessing their biocompatibility in-depth and actually finding that in their native form, certain types of MIP NPs actually elicit an immune response *in vivo* [85]. Thus far, to the best of our knowledge, no studies have been carried out to assess the environmental impact both in the short term and long term of these nanomaterials in their current non-biodegradable fashion. Fortunately, some examples have been reported in which biodegradable MIP NPs have been produced, either by exploiting whole biodegradable backbone polymers (e.g., polylactic acid, polyglycolic acid, etc.) or alternatively relying on polypeptidic backbones (hence the borderline between molecular imprinting technology and engineered peptide affinity reagents) [38,39,80].

When it comes to biodegradability, it is important to consider three aspects in particular. First, there is most likely going to be a tradeoff between the level of affinity/specificity that can be achieved and the structural and chemical stability of the nanosystem [15]. In this respect, most likely using solid-phase synthesis and/or purification technology could help in shifting the equilibrium of the production process towards the “high-affinity” fraction of the product.

**Figure 9:**
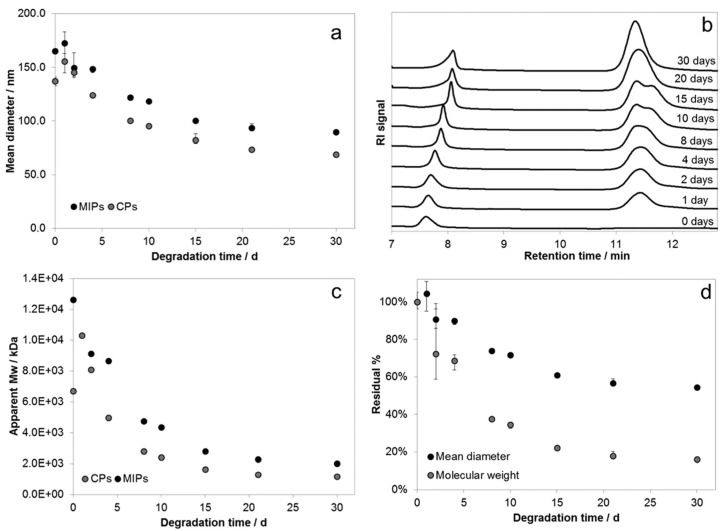
Degradation tests of the biotin-MIP NPs produced by Gagliardi *et al.*: (**a**) mean diameter decrease *vs.* time; (**b**) chromatograms for MIPs; (**c**) apparent molecular weights of degraded nanoparticles; (**d**) residual% of diameter and molecular weight of MIPs. Reproduced with permission from Gagliardi *et al.* [38].

The second aspect to take into consideration is the fact that being biodegradable means that there is a “time-constraint” parameter introduced into the equation, meaning that the MIP NPs need to exert their function for a specific application before undergoing degradation (Figure 9).

The third and final aspect to consider is related to the degradation products themselves. Ideally, they would have to belong to the GRAS category [31]. What is certainly desirable is to have a nanosystem that degrades in fragments, portions and molecules, which are not toxic to the environment or even worse to human beings.

These three aspects of course significantly complicate the development of MIP nanomaterials with appropriate biocompatibility/biodegradability aspects, but again this should be paramount in the design of every type of MIP nanosystem, both from an environmental as well as healthcare perspective.

## 4. Waste Management

In general, when it comes to MIP NPs, waste is generated during their production in terms of solvents, unreacted monomers as well as templates. Depending on the chemical nature of these species, as well as the process used for the manufacturing, this could represent an important sustainability factor to take into consideration. In many processes, diluted conditions are required; meaning that in the case of scale-up, the solvent waste produced would be quite significant. Furthermore, solution-type processes where the template is mixed together with the monomers again would result in waste. Of particular importance is the fact that most often the templates can be potent compounds (e.g., drugs or toxins active at an extremely low dosage), meaning that even if the amount used during the production process might not seem high, the potential risk to the operators and/or waste management risks can be extremely significant [11,12]. A sort of paradigm shift has taken place towards the usage of “immobilized” and recyclable templates, and this is indeed an important step towards making MIP NPs greener [13,77]. Nonetheless, in some cases, these solid-phase syntheses are performed using high-monomer concentrations; therefore, it might be beneficial to consider production strategies which could also recover unreacted monomers and perhaps exploit them in a subsequent production cycle.

Furthermore, as recently highlighted by Arabi *et al*. [11], little is known about the fate of MIPs once they are discarded after their application. This is even more relevant in the case of MIP NPs since an additional environmental nanotoxicology aspect needs to be considered [86]. Indeed, if not properly disposed of, MIP NPs could ultimately end up on land or in water bodies. As discussed previously, many of them would not be degradable and, therefore, could exert an accumulation effect, especially if the popularity and diffusion of MIP NPs escalate for diagnostic and pharmaceutical applications [87,88]. A recent study by Piletsky *et al.* actually highlighted that certain types of these nanomaterials might exhibit a significant immunogenic potential [85], and, therefore, their disposal should be performed according to specific and safe procedures. It would be interesting to ascertain whether these effects are also template dependent, but these types of investigations are still in their infancy to confirm this hypothesis.

Further developing polymeric materials which would result in biodegradable MIP NPs surely represents a very promising research avenue in terms of sustainability, but currently, there are too few examples (as well as technologies) available to actually confirm that this approach would represent a game-changer towards “greener” MIP NPs waste.

## 5. Conclusions

MIP NPs are an extremely popular and convenient format for generating imprinted polymers. Nonetheless, too little has been done in terms of sustainability and biodegradability aspects when it relates to MIP nanomaterials. Working in this direction would translate into reduced operator health risks, minimized adverse effects on the environment and increased practical applications in various fields based on advantages, such as biodegradability and eco-friendliness.

Each of the currently available methods to produce MIP NPs exhibits some disadvantages when considering the “green” aspects, be it due to solvent waste production, template toxicity/activity or energy consumption. Although certain methods might be “greener” than others, these efforts could be made pointless if they actually deter the possibility of achieving successful imprinting.

Looking at the future, we strongly believe that the way to proceed sustainably is to focus the experimental efforts on bio-based, biodegradable and GRAS materials, and to increase the investigations of biocompatibility as well as long-term exposure studies. Furthermore, it would be paramount to invest in computational approaches now more than ever considering the exponential development and access to advanced AI technologies [89,90,91]. This would ensure the production of a significant amount of data that will elucidate the safety profile of these materials (and the related waste products) for the end-users, the operators, as well as the environment, translating in turn into established “green” production standards suitable to be substantiated both at an industrial as well as research level.

## Data Availability

Not applicable.
